# Phototactic Decision-Making by Microalgae

**DOI:** 10.1103/3fry-7tsw

**Published:** 2025-11-26

**Authors:** Shantanu Raikwar, Adham Al-Kassem, Nir S. Gov, Adriana I. Pesci, Raphaël Jeanneret, Raymond E. Goldstein

**Affiliations:** 1 Laboratoire de Physique de l’École Normale Supérieure, ENS, Université PSL, CNRS, Sorbonne Université, Université Paris Cité, F-75005 Paris, France; 2 Department of Chemical and Biological Physics, Weizmann Institute of Science, Rehovot 76100, Israel; 3 Department of Physiology, Development and Neuroscience, University of Cambridge, Cambridge CB2 3DY, United Kingdom; 4 Department of Applied Mathematics and Theoretical Physics, University of Cambridge, Wilberforce Road, Cambridge CB3 0WA, United Kingdom

## Abstract

We study how simple eukaryotic organisms make decisions in response to competing stimuli in the context of phototaxis by the unicellular alga *Chlamydomonas reinhardtii*. While negatively phototactic cells swim directly away from a collimated light beam, when presented with two beams of adjustable intersection angle and intensities, we find that cells swim in a direction given by an intensity-weighted average of the two light propagation vectors. This geometrical law is a fixed point of an adaptive model of phototaxis and minimizes the average light intensity falling on the anterior pole of the cell. At large angular separations, subpopulations of cells swim away from one source or the other, or along the direction of the geometrical law, with some cells stochastically switching between the three directions. This behavior is shown to arise from a population-level distribution of photoreceptor locations that breaks front-back symmetry of photoreception.

In areas as diverse as ecology [1,2], microbiology [3], evolutionary biology, and the psychology of human behavior [4] the question arises of how individuals make decisions when confronted with competing environmental stimuli. For complex organisms with a highly developed neural system, such decision-making may involve weighing the costs and benefits of the choices along with a balance between immediate rewards and long-term consequences. The situation is less clear for aneural organisms such as plants, bacteria, and amoebas, but they appear to utilize similar mechanisms [5].

The simplest setting for decision-making clearly involves just two choices. At the scale of microorganisms there have been studies of the chemotactic response of the bacterium *Escherichia coli* to two opposing chemical stimuli [3] and the phototactic response of colonies of cyanobacteria to two light sources [6–9]. These have suggested a “summation rule” in which the addition (scalar or vector) of the two stimuli forms the basis of the decision. Similar rules have been found in the study of plants [10].

Of the many types of taxes exhibited by microorganisms—chemotaxis, phototaxis, viscotaxis, durotaxis—phototaxis is distinguished by the fact that the stimulus direction and magnitude can be changed arbitrarily fast, without the complexities of a diffusive process or intervening surfaces. In the case of algal phototaxis, a long history of studies [11–23] has shown that the light-sensing process is “line of sight” in that each cell has a photosensor that responds when directly illuminated, triggering changes in flagellar beating that produce alignment with the light. The two key ingredients for accurate phototaxis are the spinning of cells about a body-fixed axis and the directionality of the photoreceptor, achieved by a protein layer (the “eyespot”) that blocks light coming from behind the cell. Flagellar beating exhibits a rapid response to changes in light and a slower adaptation tuned to the spinning period [16,17,23,24], and cells can exhibit positive or negative phototaxis depending on light intensity and ambient biochemistry [25].

Here we report on an extensive investigation at the single cell level of phototactic decision-making by unicellular green algae. By employing the experimental setup shown in Figs. 1(a) and 1(b), in which two collimated light beams with independently adjustable intensities intersect at a prescribed angle within a dilute suspension of negatively phototactic *Chlamydomonas reinhardtii*, we track thousands of individual cells’ decisions on the choice of swimming direction. In nature, multiple light sources may arise from refraction of sunlight at a wavy air-water interface [26,27] or from light scattering by suspended particles. Our Letter is also motived by recent findings on the emergence of collective phenomena in *Chlamydomonas* populations upon multiple light sources stimulation [28]. The steady-state swimming directions are found to follow what we call the “tangent law,” an intensity-weighted average of the two light propagation vectors [29], and we show that this law is the fixed point of an adaptive model of phototaxis [20,22,23]. Studies of the response of cells to rapid changes in light direction reveal a surprisingly fast cell reorientation that can be quantitatively described as a limit of the adaptive theory. Finally, motivated by bifurcation phenomena found in certain decision-making processes [30], we examine swimming trajectories when the two lights are nearly antiparallel and find that there are three distinct subpopulations of cells: those that either (i) swim away from one source or from the other, (ii) go along the direction of the geometrical law, or (iii) exhibit stochastic switching between the first two choices. We show that this behavior arises from a population-level distribution of the location of each cell’s photoreceptor relative to the equatorial plane of the cell.

*C. reinhardtii* strain CC125 was grown axenically in trisacetate-phosphate medium, which provides it with the required nutrients. Cells were grown at 22 °C and synchronized in a light-dark cycle of 16/8h (~70 μE/(m^2^ s)) with constant shaking at 160 rpm. They were harvested in the exponential phase (~10^6^ cells/mL) when they were the healthiest and most motile. The suspension was typically diluted by a factor of 20 to avoid collective effects, placed in an open Petri dish (Falcon 353001, diameter ~3.5 cm), and kept in a dark box for 10 min before conducting experiments to ensure that all cells start from the same condition.

The experimental setup [31] consists of two collimated blue light beams (470 nm, ThorLabs COP1-A) illuminating the Petri dish located at the center of the stage of an inverted microscope (Olympus, IX83), ensuring proper control of the light directions and avoiding light gradients over the imaging field [Fig. 1(a)]. The light intensities were controlled by an LED driver (Thorlabs DC4100) through their driving currents and calibrated using a SpectraPen mini (Photon Systems Instruments). We use a 4× objective (field of view 3.7 × 3.7 mm^2^) and captured videos at 20 frames/s using a digital camera (Hamamatsu Orca Fusion-BT C15440-20UP). Image analysis used a combination of ImageJ and Matlab to track the cells; trajectories were then linked and labeled employing the Crocker-Grier algorithm [32].

Because the algal suspension is contained in a thin chamber and the lights illuminate the chamber at the very shallow angle of ~5° with respect to the plane of the stage, the swimming is effectively two dimensional. As shown in Figs. 1(a) and 1(b), the two beams are at angles ±*δ* relative to the midplane, pointing toward the positive *x* axis along the unit vectors ${\hat{v}}_{\pm }$, with *η* *=I*_±_ */I*_−_ the intensity ratio of the two beams.

*Chlamydomonas* cells, viewed from behind, spin counterclockwise around their posterior-anterior axis with frequency *f*_*r*_ = |*ω*_3_| /2*π* ~ 1.5–2 Hz [31]. Because of shading by proteins behind the photoreceptor, when a cell swims at an angle *φ* < −*δ* (region I) or *φ* > *δ* (region III) both lights illuminate the photoreceptor in the same half turn, but when |*φ* | < *δ* (region II), the photoreceptor is illuminated only by one light in each half turn.

Starting from a dark state in which cells swim randomly, highly directional swimming occurs within ~10 s of the start of illumination. The swimming paths appear as sinusoidal oscillations around linear motion because they are projections onto the *x*-*y* plane of helices. We obtain the swimming angle *φ*_*i*_ for each of typically 200–600 paths from the best-fit straight line and report ⟨φ*⟩ as the ensemble average for each choice of (*δ*, *η*), with error bars representing the standard deviation of the fitted slopes. Figure 1(c) shows that the data are well fit by the tangent law, where the swimming direction is along the unit vector ${\hat{u}}^{*}=\left(\cos\varphi^{*},\sin\varphi^{*}\right)$ defined as an intensity-weighted average of the light vectors,

(1) $${\hat{u}}^{*}=\frac{\eta {\hat{v}}_{+}+{\hat{v}}_{-}}{\left|\eta {\hat{v}}_{+}+{\hat{v}}_{-}\right|}\;\;\;\;\text{or}\;\;\;\;\text{tan}\varphi^{*}=\frac{\eta -1}{\eta +1}\tan\delta .$$

For *η* ≫ 1 (or *η* ≪ 1) the trajectory aligns with the lower (or upper) light (φ* = *δ* or φ* = −*δ*), while for equal light intensities (*η* = 1) cells swim along the *x* axis (φ* = 0), see Video S1 in Supplemental Material [31]. This law appears to be valid for half-angles *δ* as large as ~65°−70° [darkest blue inverted triangles in Fig. 1(c)], although in this situation cells do not follow the average direction as accurately, as illustrated by the increasing size of the error bars as *η* → 1 [31]. We hypothesize that such an intensity-weighted law should remain valid for more than two lights as long as the angle between the two furthest lights is small enough (2*δ* ≲ 140°).

While the result (1) makes no reference to biochemical processes in the cell, and is purely geometrical, we now show that it is a fixed point of a dynamical theory for *Chlamydomonas* phototaxis [23]. This theory combines rigid-body dynamics and an adaptive model for the angular rotation frequency *ω*_1_ around the body axis ê_1_ orthogonal to the flagellar beat plane due to asymmetries in beating of the two flagella in response to illumination of the photo-receptor. For a cell swimming in the *x*-*y* plane with a photoreceptor along ê_2_ in the cell’s equatorial plane (Fig. 4 in End Matter), and with *T=* |*ω*_3_| *t* a rescaled time, this dynamical system reduces to

(2a) $$\varphi_{T}=-P\sin T,$$

(2b) $$P_{TT}+\frac{\alpha +\beta }{\alpha \beta }P_{T}+\frac{1}{\alpha \beta }P=\frac{1}{\beta }S_{T},$$

where the subscript *T* means *∂*=*∂T*, the photoresponse variable *P =ω*_1_/ |*ω*_3_ |, and *α* and *β* are the slow flagellar adaption time and fast response time made dimensionless with |*ω*_3_|, respectively, with *α* ≫ *β* in experiments [23]. Under the assumption of additivity of light stimuli, the phototactic signal *S* = P* [*ηJ*_+_ ℋ (*J*_+_) +*J*_−_ℋ (*J*_−_)]is given by the projections $J_{\pm }=-\hat{o}\cdot {\hat{v}}_{\pm }=\sin(\varphi \mp \delta )$ sin *T* of the two lights on **ô**, the outward normal to the eyespot, where $P^{*}=\omega_{1}^{*}/|\omega_{3}$, with $\omega_{1}^{*}$ the peak turning rate around ê_1_. Here, the Heaviside functions ℋ in *S* represent the effect of eyespot shading. In the End Matter, we show that averaging over the fast timescale of cellular spinning leads to the reorientation dynamics of the swimming angle *φ* on a slow timescale *τ*,

(3) $$\varphi_{\tau }=-\lambda [\eta \sin(\varphi -\delta )+\sin(\varphi +\delta )],$$

where *λ* ∝ P*. While (3) depends on cellular parameters via *λ*, its steady-state solution φ* is the tangent law (1).

The dynamics (3) has a Lyapunov function *V*(*φ*) in the sense that *λ*^−1^*dφ*/*dτ*, = −*dV/dφ*, with

(4) V(φ)=−[ηcos(φ−δ)+cos(φ+δ)].

A simple calculation shows that $V=-{\hat{e}}_{3}\cdot \left(\eta {\hat{v}}_{+}+{\hat{v}}_{-}\right)$, the projection of the total light vector onto the anterior pole of the cell, and that *V* is a minimum at φ*; for negative phototaxis, a cell chooses a direction that minimizes the average light falling onto its anterior pole. As shown in the End Matter, for positive phototaxis the tangent law still holds, with the selected angle φ* shifted by *π*, and the Lyapunov function is −*V*, which is the projection of light on the posterior pole of the cell.

Moving on from the steady-state results in Fig. 1, we study how cells respond to a rapid switch in illumination between lights. Figure 2(a) shows cellular trajectories for 2*δ* = 70° (Video S2 in [31]). These turns are quantified through the angle *ξ*(*T*) between the local unit tangent $\hat{t}$ to the trajectory and the new light direction. Results for five different light intensities are shown in Fig. 2(b), which illustrates that complete reorientation occurs within just over one period of rotation (*T* ≃ 2*π*), with a modest dependence on light intensity. Within the adaptive theory, this rapid orientation corresponds to P* ~ 1. When *α* ≫ *β* and *αβ* ~ 1 as found in prior work [23], the dominant balance in (2b) gives *P* ≃ *S* and thus *ξ*_*T*_ =P* sin *ξ*ℋ (sin *T*)sin^2^*T*. In terms of the shift $\tilde{T}=T-T_{0}$ relative to the switching time *T*_0_, we find

(5) $$\cos\xi (\tilde{T})=\tanh\left\{P^{*}[2\tilde{T}-\sin2\tilde{T}]/4-C\right\},$$

where *C* = ln [tan (*ξ*_0_/2)] and *ξ*_0_ is the initial angle. To compare with the experimental data, we note that when the lights are switched the cells are at random phases of their wiggly motion, and we average (5) over a uniformly distributed angle *ξ*_0_ ∈ [−110° − *ξ*_*i*_, −110° +*ξ*_*i*_], where *ξ*_*i*_ = 22°, as measured directly (see Supplemental Material, Fig. S7 [31]). The result shown in Fig. 2(b) matches the data well, with P* ≃ 1.4 as the sole fitting parameter, reflecting the accuracy of this simplified model.

The three types of trajectories described in the Introduction, shown in Fig. 3(a) for 2*δ* = 162° (Video S3 in [31]), are quantified in Fig. 3(b) by partitioning each into 3s segments (~5 body rotations) and finding the average orientation angle *φ* for each. The probability distribution *Q*(*φ*) exhibits three peaks corresponding to swimming away from either light or along the *x* axis.

The theory discussed thus far cannot account for stable negatively phototactic swimming away from one light or the other when the photoreceptor is in the cell’s equatorial plane; the only stable fixed point is φ* = 0. But if the photoreceptor is displaced from the equator by an angle γ *<* 0, such motion is stable; due to photoreceptor shielding by the eyespot, a cell swimming away from one light must rotate through γ to sense the other. Such rotations may arise from flagellar beating jitter [23].

Using a method based on light reflection from the eyespot [31,33], we measured the probability distribution *q*(*h*) of eyespot displacements *h* from the midplane of 204 cells, and we show in Fig. 3(c) that it is well fit by a Gaussian with mean 0.15 μm and standard deviation 1.0 μm (positive values correspond to eyespots closer to the flagella). A significant subpopulation has eyespots displaced more than the eyespot diameter from the midline.

It follows from the considerations above that during the finite duration of an experiment, cells with eyespots far below the midplane will have not had sufficient time to fluctuate enough to see the other light and thus will swim away from one light or the other. For those with eyespots far above the midline, direct swimming away from either light is unstable, and those with intermediate positions can exhibit stochastic hopping between the three choices. To test the hypothesis that eyespot displacement is the origin of the distribution in Fig. 3(b), we generalize the model by writing a Langevin equation for the orientation with a shifted photoreceptor. The dynamical system is *φ*_*T*_ = −*P* sin *T* +*ξ*(*T*), where $\langle \xi (T)\rangle =0,\langle \xi (T)\xi \left(T^{\prime }\right)\rangle =2{\tilde{D}}_{r}\delta \left(T-T^{\prime }\right)$, ${\tilde{D}}_{r}=D_{r}/\left|\omega_{3}\right|$ is the scaled rotational diffusion constant, and the projections are

(6) $$J_{\pm }=\cos\gamma \sin(\varphi \mp \delta )\sin T-\sin\gamma \cos(\varphi \mp \delta ),$$

where *h = R* sin *γ*, with *R=* 5 μm the cell radius. Details of numerical studies of this stochastic phototaxis are given in the End Matter. Figure 3(d) shows the resulting probability distribution function of trajectory angles, which strongly resembles the experimental one. We find that the peaks are associated with different subpopulations of the cells. Those with a large negative offset angle *γ* move directly away from one or the other light, while those with a strongly positive offset follow the tangent law. Cells with an offset close to the middle of these two extremes stochastically switch their trajectories.

By defining an effective free energy *F(φ*) = − ln *Q* from the measured distributions in the numerical computations, we find an underlying bifurcation in the decision-making process. Simulations in which both *δ* and *h* are fixed show [Fig. 3(e)] a transition from a single minimum in *F* at small *h* to a double-well structure at larger *h*. Thus, the observed three-peak distribution function in Fig. 3(d), obtained for an ensemble of cells with different eyespot offsets, reflects a superposition of one- and two-minimum free energies. Further evidence for the existence of an underlying bifurcation at large angles is found in the increasing scale of fluctuations around the tangent law seen in Fig. 1(c) for large *δ* as *η* → 1 [31].

The approach presented here shows how mechanistic insights into biological decision-making can be gained by going beyond typical forms of stimuli found in natural contexts. Yet, since a continuously varying natural light field can be represented as a superposition of discrete sources, our results suggest that the generalization of our model to one with *N* → ∞ lights would imply that negatively (positively) phototactic cells would swim away from (toward) the brightest spot, thus providing a link between line-of-sight and gradient-climbing approaches [34–36]. Issues for further study involve the possibility of a dynamic bifurcation [37] when the lights are point sources, not at infinity, and their apparent positions slowly vary as cells swim [30], as well as the effects of longer-term adaptations associated with photosynthesis [38,39]. Finally, we may ask whether multicellular organisms related to *Chlamydomonas* and yet having no central nervous system [40] would follow the same decision-making rules.

## Supplementary Material

SM

End Matter

## Figures and Tables

**Fig. 1:**
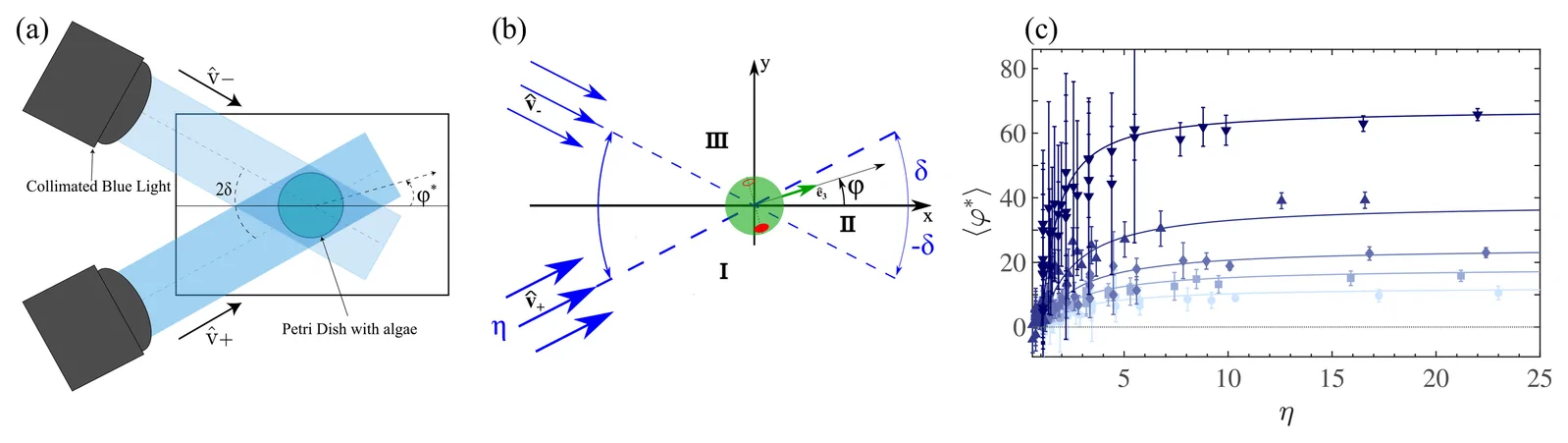
Experimental results. (a) Setup: two collimated lights shine toward the *x* axis at angles ± *δ*, the lower with intensity *η* relative to the upper. (b) A swimming cell whose axis ê_3_ is at an angle *φ* with respect to the *x* axis. Eyespot at two instants in time separated by a half cycle is shown as a solid and open red ellipse. (c) Swimming angle as a function of intensity ratio *η* for *δ* = 12.5°, 18.45°, 24.74°, 38.4°, and 67.6° increasing upward, along with the theoretical prediction (1) for each value of *δ*.

**Fig. 2:**
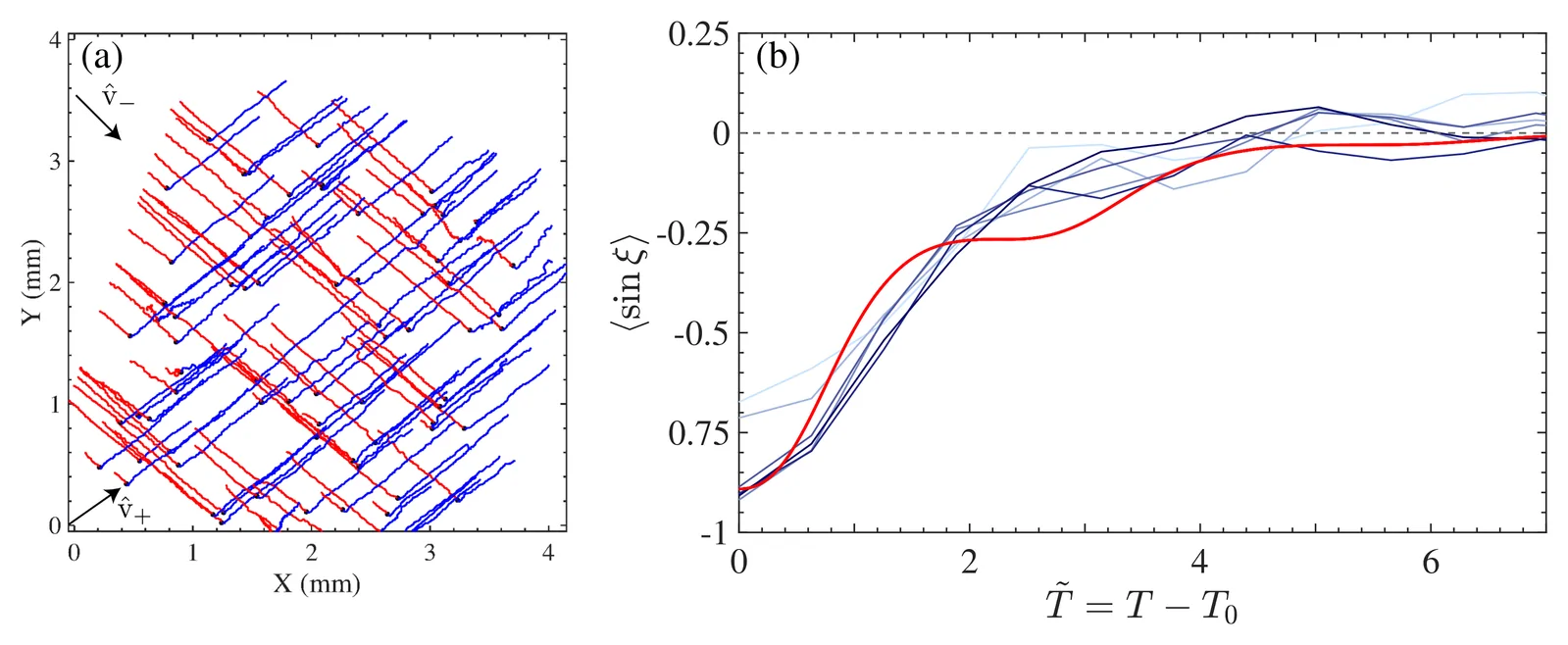
Reorientation dynamics. (a) Trajectories during a switch in light direction from ${\hat{v}}_{-}(\text{red})$ to ${\hat{v}}_{+}(\text{blue})$ for *η* = 1. Black circle indicates time of switch. (b) Reorientation angle *ξ* during phototurn at intensities *I* = 1.8, 3.7, 9.1, 17.7, 24.8, 33.9 W/m^2^, color coded from light to dark blue compared to (5) (red) averaged over initial phase of motion.

**Fig. 3:**
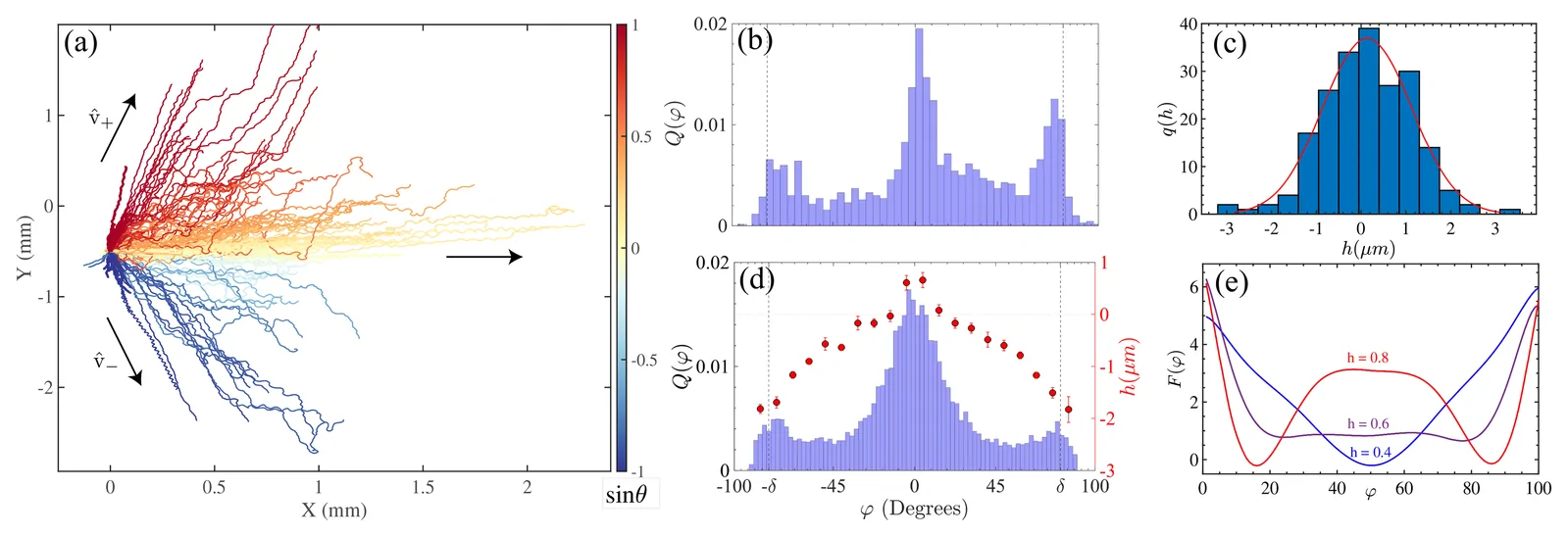
Phototaxis at large angular light separation. (a) Trajectories for 2*δ* = 162° and *I*_±_ = 3.7 W/m^2^, showing negative phototaxis, following tangent law, and stochastic switching between directions. Trajectories are color coded by orientation of their end-to-end vectors relative to the *x* axis. (b) Probability distribution *Q*(*φ*) of trajectory angles for *I*_±_ = 3.7 W/m^2^. (c) Distribution of eyespot offsets along with Gaussian fit. (d) Numerical *Q*(*φ*) from model of stochastic phototaxis incorporating distribution of eyespot offsets from equator. (e) Effective free energy as a function of eyespot offset for 2*δ* = 162°.

## Data Availability

The data that support the findings of this article are openly available [41].
