# Supplementary material for: Phototactic Decision-Making by Microalgae
Source: Phys Rev Lett. Author manuscript; Available in PMC 2026 Sep 12. (PMC7619487; doi:10.1103/3fry-7tsw)
Supplement: Supplementary file 1 [file EMS218213-supplement-End_Matter.pdf]

[25] Z. Wang and A. C. H. Tsang, Intermediate light adaptation induces oscillatory phototaxis switching and pattern formation in Chlamydomonas, Proc. Natl. Acad. Sci. U.S.A. **122**, e2425369122 (2025).

[26] R. L. Snyder and J. Dera, Wave-induced light-field fluctuations in the sea, J. Opt. Soc. Am. **60**, 1072 (1970).

[27] J. Wei, M. R. Lewis, R. Van Dommelen, C. J. Zappa, and M. S. Twardowski, Wave-induced light field fluctuations in measured irradiance depth profiles: A wavelet analysis, J. Geophys. Res. Oceans **119**, 1344 (2014).

[28] A. L'Homme, A. Lahlou, S. Bujaldon, T. Le Saux, B. Bailleul, N. Desprat, and R. Jeanneret, Light-induced phase separation with finite wavelength selection in photophobic microalgae, Phys. Rev. Lett. **135**, 148401 (2025).

[29] A much earlier experimental study of the unicellular organism *Euglena gracilis* examined phototaxis in the presence of two lights, with adjustable intensities but a single angular separation, and found similar phenomenology to that reported here, described with an empirical fit to the data, D. P. Häder, M. Lebert, and M. R. Di Lena, New evidence for the mechanism of phototactic orientation of Euglena gracilis, Curr. Microbiol. **14**, 157 (1986).

[30] V. H. Sridhar, L. Li, D. Gorbonos, M. Nagy, B. R. Schell, T. Sorochkin, N. S. Gov, and I. D. Couzin, The geometry of decision-making in individuals and collectives, Proc. Natl. Acad. Sci. U.S.A. **118**, e2102157118 (2021).

[31] See Supplemental Material at http://link.aps.org/supplemental/10.1103/3fry-7tsw for further experimental results.

[32] J. C. Crocker and D. G. Grier, Methods of digital video microscopy for colloidal studies, J. Colloid Interface Sci. **179**, 298 (1996).

[33] N. Isogai, R. Kamiya, and K. Yoshimura, Dominance between the two flagella during phototactic turning in Chlamydomonas, Zool. Sci. **17**, 1261 (2000).

[34] C. R. Williams and M. A. Bees, Photo-gyrotactic bioconvection, J. Fluid Mech. **678**, 41 (2011).

[35] L. de Andres-Bragado, C. Mazza, W. Senn, and S. G. Sprecher, Statistical modelling of navigational decisions based on intensity versus directionality in Drosophila larval phototaxis, Sci. Rep. **8**, 11272 (2018).

[36] M. L. Zhu, K. J. Herrera, K. Vogt, and A. Bahl, Navigational strategies underlying phototaxis in Drosophila larvae, J. Exp. Biol. **224**, jeb242428 (2021).

[37] N. R. Lebovitz and A. I. Pesci, Dynamic bifurcation in Hamiltonian systems with one degree of freedom, SIAM J. Appl. Math. **55**, 1117 (1995).

[38] M. M. Moses, D. W. Morris, and W. Qin, Greener on the other side of the fence: Density-dependent habitat selection by a unicellular alga, Evol. Ecol. Res. **15**, 1 (2013), https://www.scopus.com/inward/record.uri?eid=2-s2.0-84894144532&partnerID=40&md5=ee48fab54c523fc0cd948260afcad70c.

[39] J. Arrieta, A. Barreira, M. Chioccioli, M. Polin, and I. Tuval, Phototaxis beyond turning: Persistent accumulation and response acclimation of the microalga Chlamydomonas reinhardtii, Sci. Rep. **7**, 3447 (2017).

[40] R. E. Goldstein, Green algae as model organisms for biological fluid dynamics, Annu. Rev. Fluid Mech. **47**, 343 (2015).

[41] S. Raikwar, A. Al-Kassem, N. S. Gov, A. I. Pesci, R. Jeanneret, and R. E. Goldstein, Phototactic decision-making by microalgae, Zenodo, 2025, 10.5281/zenodo.15051634.

End Matter

The complete model introduced earlier [23] consists of the coupled dynamics of the Euler angles (ϕ, θ, ψ) describing rigid-body motion and the adaptive dynamics of the angular rotational velocity ω_1 about the axis $\hat{\mathbf{e}}_1$ that is orthogonal to the plane of the flagellar beating. In a system of units scaled by the magnitude of the spinning angular velocity $|\omega_3|$ about the cellular axis $\hat{\mathbf{e}}_3$ that lies in the plane of beating and is equidistant to the bases of the two flagella (Fig. 4), the dynamics takes the form

$$\phi_T = (\hat{P} + P)\frac{\sin\psi}{\sin\theta}, \tag{A1a}$$

$$\theta_T = (\hat{P} + P)\cos\psi, \tag{A1b}$$

$$\psi_T = -1 - (\hat{P} + P)\frac{\sin\psi\cos\theta}{\sin\theta}, \tag{A1c}$$

$$\beta P_T = S - H - P, \tag{A1d}$$

$$\alpha H_T = S - H, \tag{A1e}$$

where $\hat{P} = \hat{\omega}_1/\omega_3$ and $P = \omega_1/\omega_3$ are the scaled intrinsic and photoresponse rotation frequencies around $\hat{\mathbf{e}}_1$, respectively, H is the hidden variable responsible for adaptation, and α and β are the scaled adaptation and response times for the photoresponse system.

In earlier work [23], we found two intrinsic timescales of relevance to a phototactic turn: a short one associated with the cell's spinning motion about the axis $\hat{\mathbf{e}}_3$ and a longer one for the reorientation of $\hat{\mathbf{e}}_3$ toward a collimated light

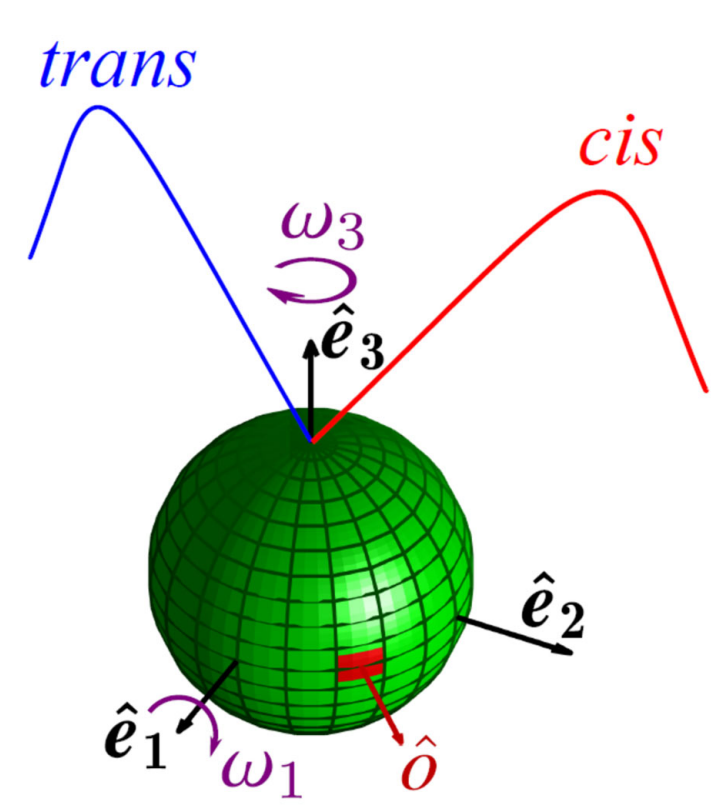

FIG. 4. Coordinate system of *Chlamydomonas*, from Ref. [23]. Eyespot is shown as red square.

beam. In this section, we provide a systematic derivation of the averaged dynamics based on the presumed smallness of the photoresponse variable P, focusing first on the case of a single light source pointing along $-\hat{\mathbf{e}}_x$ and then generalizing to the geometry of the two light sources considered in the main text. To make analytical progress, we ignore the small helical component of the motion, take the photoreceptor to be along $\hat{\mathbf{e}}_2$, and fix the Euler angle $\theta = \pi/2$ to neglect the small out-of-plane motion. Finally, with P small, we may approximate the third Euler angle by $\psi = -T$ since the time evolution of ψ is dominated by rotations around $\hat{\mathbf{e}}_3$. This yields the simplified model for the Euler angle ϕ,

$$\phi_T = -P \sin T, \tag{A2a}$$

$$\beta P_T = S - H - P, \tag{A2b}$$

$$\alpha H_T = S - H. \tag{A2c}$$

Under the adaptive dynamics (A1d) and (A1e), for positive S a temporal maximum $P_{\max}$ in P requires $S - H - P_{\max} = 0$, and since $H \geq 0$ we obtain that the maximum possible value of P is $P^* = S$. This can be seen explicitly for square-wave signals [23], but holds more generally. This result allows us to bypass the need to calibrate a relationship between the light intensity and the photoresponse variable, and instead measure the signal amplitude by the maximum photoresponse (ω_1) induced.

We set P^*, the scaled maximum magnitude of the photoresponse, to be

$$P^* = -m\epsilon, \tag{A3}$$

with $m = \pm 1$ for positive (negative) phototaxis and the small parameter is $0 < \epsilon \ll 1$. Then the signal is

$$S = m\epsilon \cos\phi f(T) \quad \text{with} \quad f(T) = \sin T \mathcal{H}(\sin T). \tag{A4}$$

Noting that (A2b) and (A2c) can be combined into a single second-order equation for P [23],

$$\mathcal{L}P \equiv P_{TT} + \frac{(\alpha+\beta)}{\alpha\beta} P_T + \frac{1}{\alpha\beta} P = \frac{1}{\beta} S_T, \tag{A5}$$

we now seek a perturbative solution to the dynamics (A2) and (A5) in powers of ϵ.

As the intrinsic rate of variation of P given by α and β is $\mathcal{O}(1)$, it is natural to assume that when coupled to the slow variable ϕ the dynamical variables ϕ and P can be written as functions of two variables: a fast one (T) and a slow one ($\tau = \epsilon T$). Thus, assuming the forms $\phi(T, \tau)$, etc. we have the rule

$$\frac{d}{dT} \to \frac{\partial}{\partial T} + \epsilon \frac{\partial}{\partial \tau}. \tag{A6}$$

If we now propose perturbative expansions of the form

$$\phi(T,\tau) = \phi^{(0)}(T,\tau) + \epsilon\phi^{(1)}(T,\tau) + \cdots, \tag{A7a}$$

$$P(T,\tau) = P^{(0)}(T,\tau) + \epsilon P^{(1)}(T,\tau) + \cdots, \tag{A7b}$$

then at $\mathcal{O}(\epsilon^0)$ we find

$$P^{(0)} = 0 \quad \text{and} \quad \frac{\partial \phi^{(0)}(T,\tau)}{\partial T} = 0, \tag{A8}$$

which implies that $\phi^{(0)}$ depends only on the slow variable τ. This solution corresponds to the fixed point of the underlying adaptive dynamics in the absence of a stimulus.

At $\mathcal{O}(\epsilon^1)$ we find

$$\frac{\partial \phi^{(1)}}{\partial T} = -\frac{\partial \phi^{(0)}}{\partial \tau} - \sin T P^{(1)}, \tag{A9a}$$

$$\mathcal{L}P^{(1)} = \frac{m}{\beta} \cos[\phi^{(0)}(\tau)] \frac{df(T)}{dT}. \tag{A9b}$$

Note that the rhs of (A9a) does not depend on $\phi^{(1)}$ and thus functions as a forcing term. Moreover, at this order, the right-hand side of (A9b) is a function of T, with τ playing the role of a parameter.

A bounded solution to (A9a) requires implementing the solvability condition that the rhs of (A9a) be orthogonal to the null space of the lhs operator. That null space is a constant, leading to the result

$$\frac{1}{2\pi} \int_0^{2\pi} \frac{\partial \phi^{(0)}}{\partial \tau} dT = \frac{\partial \phi^{(0)}}{\partial \tau} = -\frac{1}{2\pi} \int_0^{2\pi} \sin T P^{(1)}(T,\tau) dT. \tag{A10}$$

In the multiple-scale analysis, reorientation toward the light is slow, and at each value of ϕ the flagellar response has time to reach a quasisteady state in which $P^{(1)}$ is the same from one rotation to the next. Hence, the rhs of (A10) is proportional to the discrete Fourier transform with respect to T of that steady-state response at the cellular rotation frequency. This transform is given by the Green's function of the damped harmonic oscillator whose operator is $\mathcal{L}$.

A great simplification of this analysis can be made in light of our earlier work [23], which showed that representative values of the dimensional time constants in (A2b) and (A2c) are $\alpha \simeq 7 \gg \beta \simeq 0.14$. These values imply that the dominant balance of terms in the second-order equation (A5) is $P_T \sim S_T$ and hence $P \sim S$; the photoresponse is directly proportional to the signal. This yields

$$P^{(1)}(T,\tau) \simeq m \cos\phi^{(0)}(\tau) f(T). \tag{A11}$$

Shifting to $\varphi^{(0)} = \phi^{(0)} - \pi/2$, we obtain from (A10)

$$\frac{\partial \varphi^{(0)}}{\partial \tau} = m\lambda \sin\varphi^{(0)}, \tag{A12}$$

where $\lambda(\alpha, \beta) = 1/4$ in the limit considered here. With initial condition $\varphi_0 = \varphi^{(0)}(0)$ and returning to dimensional units, the reorientation to a single light shining toward the positive x axis follows

$$\varphi^{(0)}(t) = 2\tan^{-1}\left[\tan\left(\frac{\varphi_0}{2}\right)e^{m\lambda\omega_1^* t}\right]. \qquad \text{(A13)}$$

Intuitively, the turning rate in (A13) is proportional to the maximum angular velocity ω_1^* achievable by the cell, attenuated by the factor of λ due to the geometry of light projection on the eyespot of a rotating cell. For positive phototaxis ($m = +1$), $\varphi \to \pi$ as $\tau \to \infty$ (the cell swims along $-\hat{\mathbf{e}}_x$, antiparallel to the light), whereas $\varphi \to 0$ as $\tau \to \infty$ for negative phototaxis ($m = -1$), as the cell swims parallel to the beam.

The generalization of (A12) to the case of two lights at angles $\pm\delta$, involves the signal defined in the main text, $S = P^*[\eta J_+\mathcal{H}(J_+) + J_-\mathcal{H}(J_-)]$, with the light projections $J_\pm = -\hat{\mathbf{o}} \cdot \hat{\mathbf{v}}_\pm = \sin(\varphi \mp \delta)\sin T$. This leads directly to the dynamics

$$\frac{\partial\varphi^{(0)}}{\partial\tau} = m\lambda\left[\eta\sin(\varphi^{(0)} - \delta) + \sin(\varphi^{(0)} + \delta)\right]. \qquad \text{(A14)}$$

The general Lyapunov function is

$$V_m(\varphi) = m[\eta\cos(\varphi - \delta) + \cos(\varphi + \delta)]. \qquad \text{(A15)}$$

Here, $V_m = m\hat{\mathbf{e}}_3 \cdot (\eta\hat{\mathbf{v}}_+ + \hat{\mathbf{v}}_-)$ is the projection of the total light vector onto the posterior pole ($m = 1$) or anterior pole ($m = -1$) of the cell. In either case, the tangent law (1) holds, with $|\phi^*| < \pi/2$ for negative phototaxis and $|\phi^*| > \pi/2$ for positive phototaxis, and V_m is a minimum at the relevant φ^*. The similarity of dynamics for positive and negative phototaxis extends to the case of large separation angles δ, where positively phototactic cells also display a three-peaked distribution with subpopulations of cells sorted by eyespot location. This behavior is consistent with earlier results on *Euglena* [29], whose eyespot is very close to the anterior of the cell.

In numerical studies of stochastic phototaxis, we typically simulate $N = 10^3$ realizations of the dynamics, in which for each trajectory the eyespot location angle γ is chosen from a Gaussian distribution with parameters taken from experiment [Fig. 3(c)]. The time stepping was implemented in Matlab using the Euler-Maruyama method for the phototactic dynamics in the limit $P \simeq S$ as above. Trajectories typically lasted 60 rotational periods, and after discarding the first five periods to allow transients to decay away, the subsequent motion was analyzed as in the experiments by fitting straight lines to segments of five rotational periods and finding the average orientation angle φ for each, after which the data were aggregated into the histogram displayed in Fig. 3(d).
