# Supplementary material for: Phototactic Decision-Making by Microalgae
Source: Phys Rev Lett. Author manuscript; Available in PMC 2026 Sep 12. (PMC7619487; doi:10.1103/3fry-7tsw)
Supplement: Supplementary file 2 [file EMS218213-supplement-SM.pdf]

Supplemental Material:
Phototactic Decision-Making by Micro-Algae

Shantanu Raikwar,[1] Adham Al-Kassem,[1] Nir S. Gov,[2,3] Adriana I. Pesci,[4] Raphaël Jeanneret,[1] and Raymond E. Goldstein[4]

[1]*Laboratoire de Physique de l'École Normale Supérieure, ENS, Université PSL, CNRS, Sorbonne Université, Université Paris Cité, F-75005 Paris, France*
[2]*Department of Chemical and Biological Physics, Weizmann Institute of Science, Rehovot 76100, Israel*
[3]*Department of Physiology, Development and Neuroscience, University of Cambridge, Cambridge CB2 3DY, United Kingdom*
[4]*Department of Applied Mathematics and Theoretical Physics, University of Cambridge, Wilberforce Road, Cambridge CB3 0WA, United Kingdom*

(Dated: October 5, 2025)

S1. FOURIER ANALYSIS OF CELL TRAJECTORIES IN SINGLE-LIGHT EXPERIMENTS

To understand in detail the effect of light intensity on the swimming of the cells, we first studied the trajectories in response to a single collimated light beam. Experiments were performed using a $\times 4$ objective at 90 fps in order to obtain well resolved Fourier spectra of the motion. Because cell trajectories present modulations at frequencies below 0.5 Hz when cells follow a single light direction (Fig. S1(a), solid blue line), we first applied a Savitzy-Golay (SG) filter to the tracks in order to define properly the underyling mean motion around which are fluctuations associated with the the helical motion of the cells. The parameters of the SG filter were fixed to window size of 49 frames and polynomial order as 1. The SG filter then yields a mean trajectory (dashed black line in Fig. S1(a)) around which we can analyze the fluctuations of the filtered path, as shown in Fig. S1(b). The Fourier transforms for several light intensities shown in Figs. S1(c,d) are obtained by first trimming all trajectories at a given intensity to a common duration of 1300 frames, computing the FFTs of each, and then averaging those spectra. We observe a clear peak at $1.5 - 2$ Hz, common to all intensities, that corresponds to the frequency of helical swimming. Interestingly, the structure of the spectra above 2 Hz appears to depend on the light intensity; at low intensity there is a small peak (or "shoulder") between 3.5 and 4 Hz that disappears as the light intensity is increased, while other peaks appear at just below 6 and 8 Hz. Other peaks are also present at larger frequencies (between 10 and 20 Hz) as shown in Fig. S1(c). The biological explanation for these high-frequency peaks and their dependence on light intensity remains unclear.

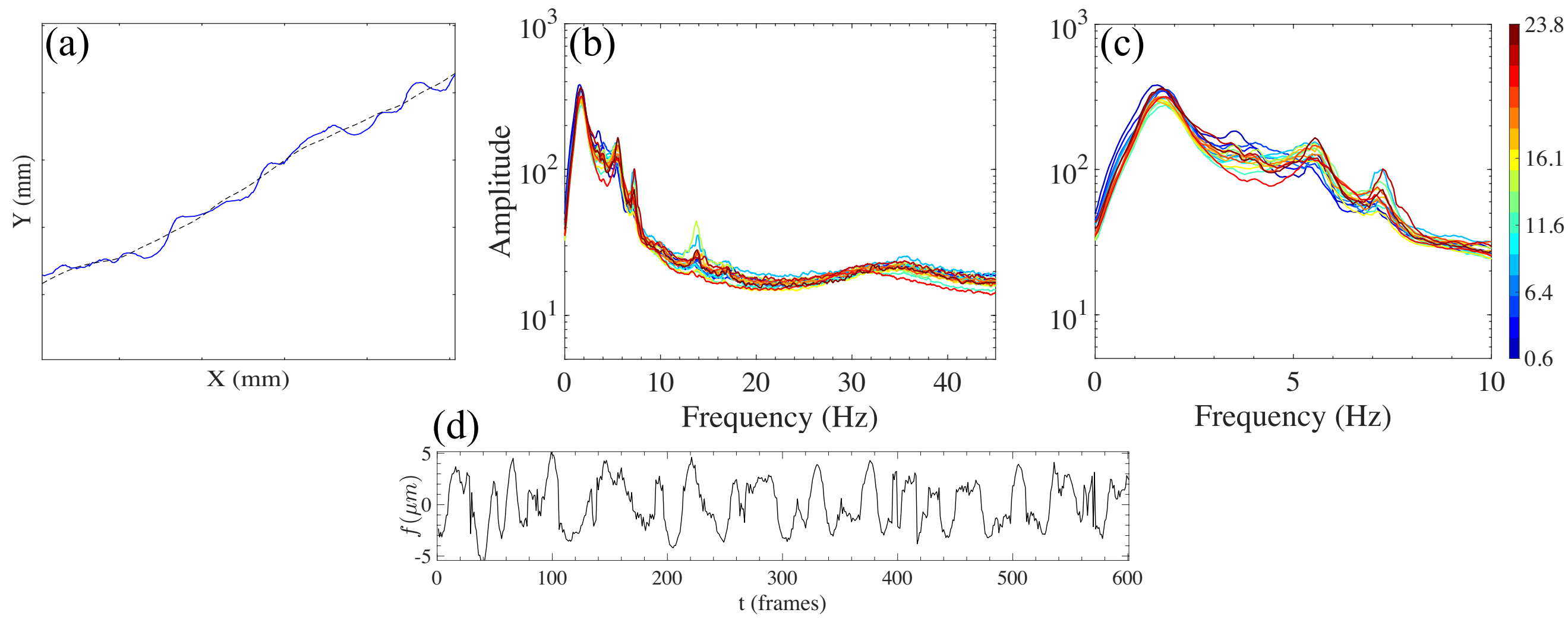

FIG. S1. Analysis of swimming trajectories. (a) Typical alga trajectory (solid blue) with its mean computed from the SG Filter (dotted black). (b) Fourier spectra for different light intensities (c)Enlargement of the first 10 Hz of the in (a) around the mean trajectory computed from the SG spectra shown in (c) (colorbar in W/m^2). (d)Fluctuations of the trajectory shown filter.

The broad peaks seen between 30 and 40 Hz, corresponding to the small, fast fluctuations visible in Fig. S1(d), arise from detection noise of the positions of cells.

S2. TANGENT LAW

A. Current-to-Power conversion

The LED driver controls the electrical current sent to the device. To calibrate the power transmitted by the LED, we used a power meter (Thorlabs controller PM100D with S130VC power sensor) that is held perpendicular to the collimated light beam, at the center of the stage, where we image the algae. We perform a linear fit to the plot of power versus current, the slope of which yields the calibration coefficient that is used in converting the ratio i_+/i_- of the electrical currents to a ratio of light power or light intensity $\eta = P_+/P_- = I_+/I_-$. This measurement is repeated over all experiments with different angles. An example of a calibration curve is shown in Fig. S2.

For information, with our blue LED sources the conversion of light intensity in $\mathrm{W/m^2}$ to $\mu\mathrm{E/m^2/s}$ is given by: $1\mathrm{W/m^2} = 3.96\mu\mathrm{E/m^2/s}$.

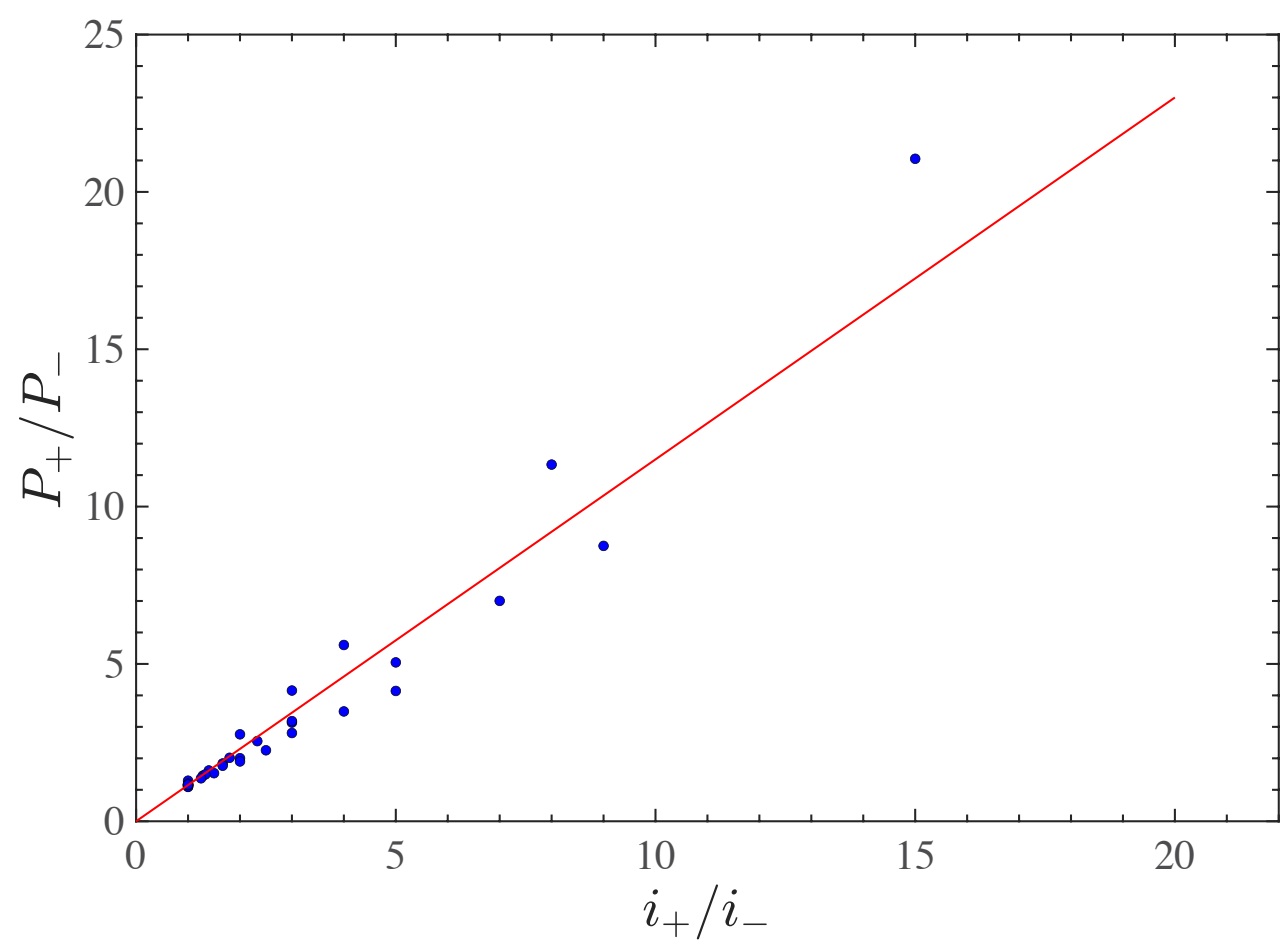

FIG. S2. Calibration curve for $2\delta = 49.5°$ with the linear fit shown in red (here $P_+/P_- = 1.15\, i_+/i_-$).

B. Experimental determination of the angles for the tangent law

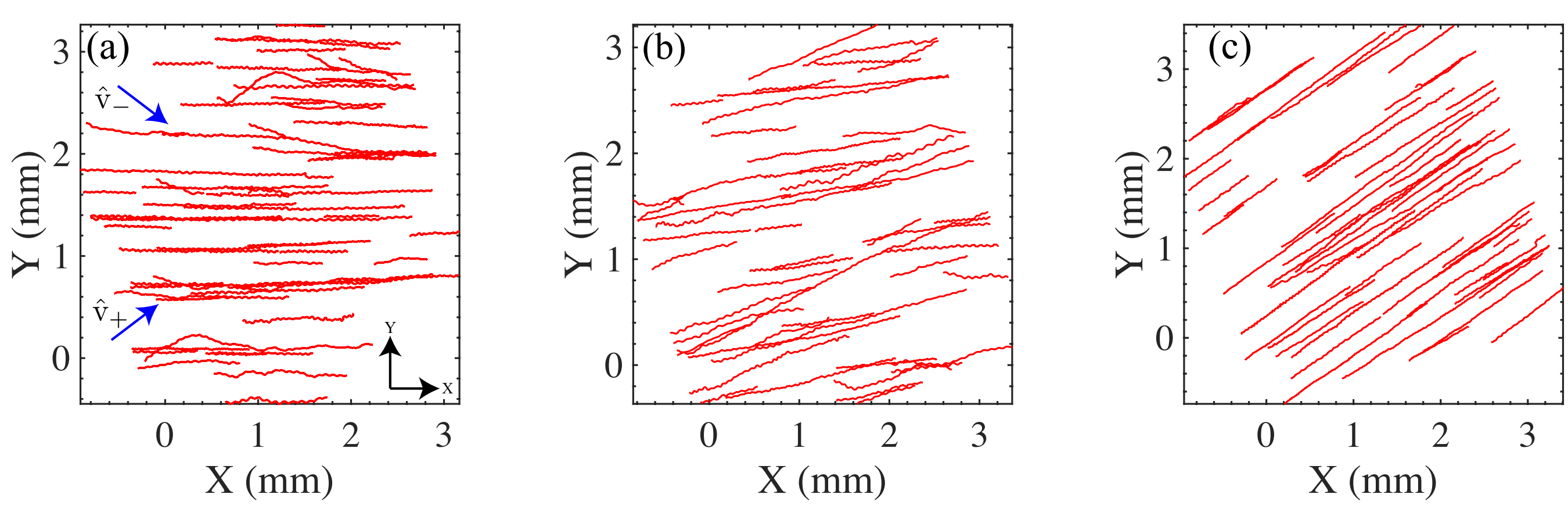

FIG. S3. Trajectories for $2\delta = 76.8°$ under different light intensity ratios: (a) $\eta = 0.74$, (b) $\eta = 2.9$, (c) $\eta = 16.6$.

To obtain the tangent law, two series of experiments were performed for each fixed position of the two lights. We first performed tracking experiments with each light turned on independently, which allows us to get an accurate value of the angle 2δ between the lights in the frame of the camera. This relies on the fact that cells do follow accurately the light propagation direction when a single light source is on. The ensemble average of the swimming direction of many cells, each obtained by a linear fit to its trajectory, is then used to define the light propagation directions. We then performed experiments with the two lights turned on to extract the ensemble average angle of swimming $\langle \varphi^* \rangle$ as a function of η, obtained also by a linear fit to the trajectories. Figures S3(a-c) show representative trajectories of the cells for a fixed $2\delta = 76.8°$ at different values of η, illustrating how the swimming angle φ^* changes as η increases.

C. Normalized Tangent Law

In order to better show the experimental data points for small values of η, we plot in Fig. S4 the normalized data $\tan(\varphi^*)/\tan(\delta)$ as a function of η for $0.5 < \eta < 5$ and for all δ. The data points are seen to follow well the theoretical prediction

$$\frac{\tan \varphi^*}{\tan \delta} = \frac{\eta - 1}{\eta + 1}. \tag{S1}$$

This good agreement is confirmed by computing the reduced chi-squared,

$$\chi^2_\nu = \frac{1}{(N-1)} \sum_{i=1}^{N} \frac{\text{dist}_i^2}{\sigma_i}, \tag{S2}$$

where dist_i is the distance between each data point i and the theoretical prediction, σ_i is the variance of each data point and N the total number of data points. We obtain $\chi^2_\nu = 0.89$, indicating a good fit.

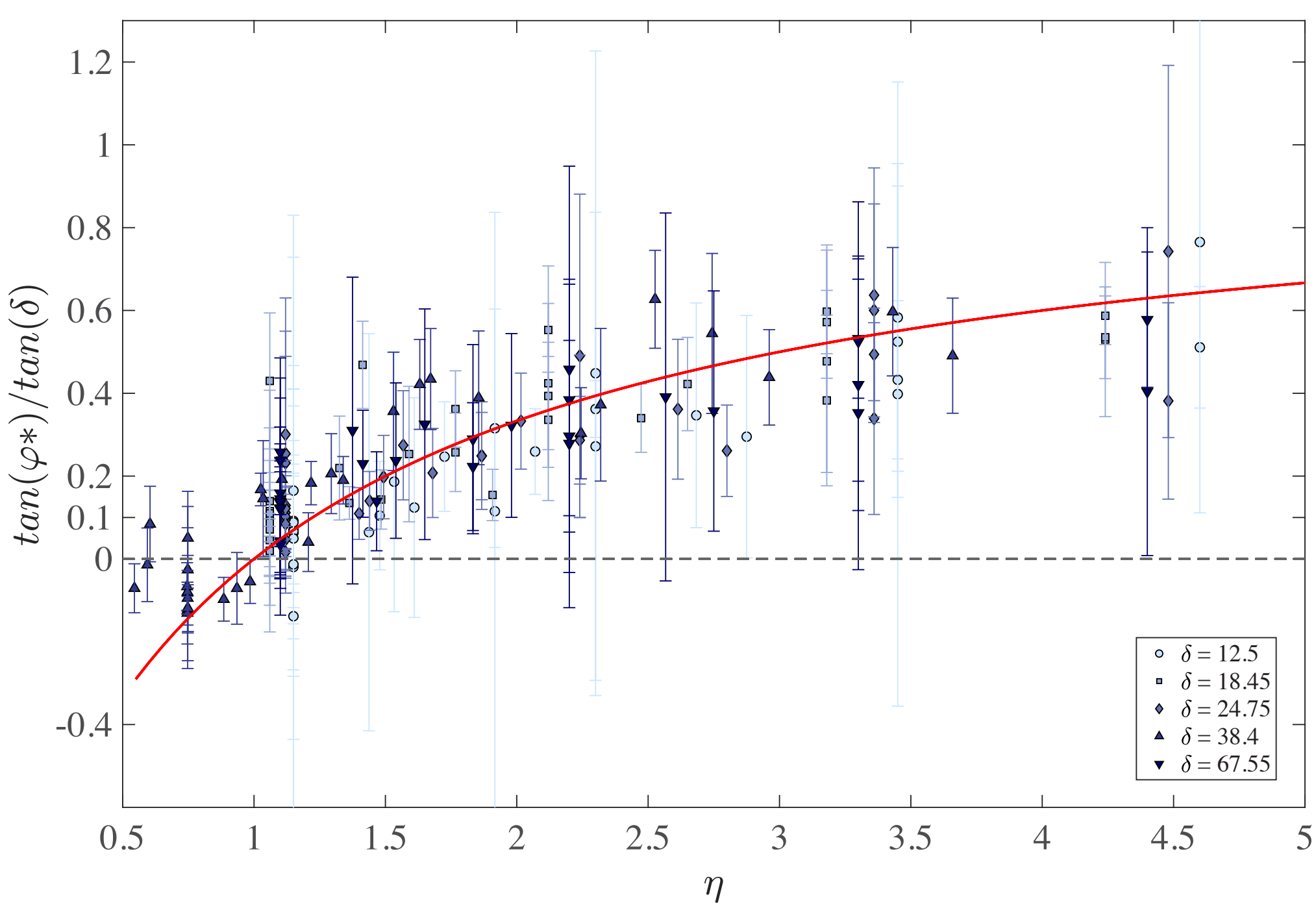

FIG. S4. Normalized data $\tan(\varphi)/\tan(\delta)$ as a function of η for $0.5 < \eta < 5$ and for all δ, showing good agreement with the theoretical prediction Eq. S1.

D. Analysis of the standard deviation of φ^*

As the angle between the two lights is increased to $2\delta \approx 135°$, we observed that the fluctuations in cell swimming direction drastically increased as η gets closer to 1 (Fig. S5). Although the tangent law can here still be considered as valid on average, the ability of cells to follow the intensity-weighted average light direction is impeded at such large angles. This is a first sign that a bifurcation will be observed for larger angles, as we see for $2\delta = 162°$. As discussed in the main text, the three-peak distribution of trajectory angles arises from a superposition of the properties of subpopulations of cells. For this reason, there should be no sharp transition or true bifurcation as δ is increased.

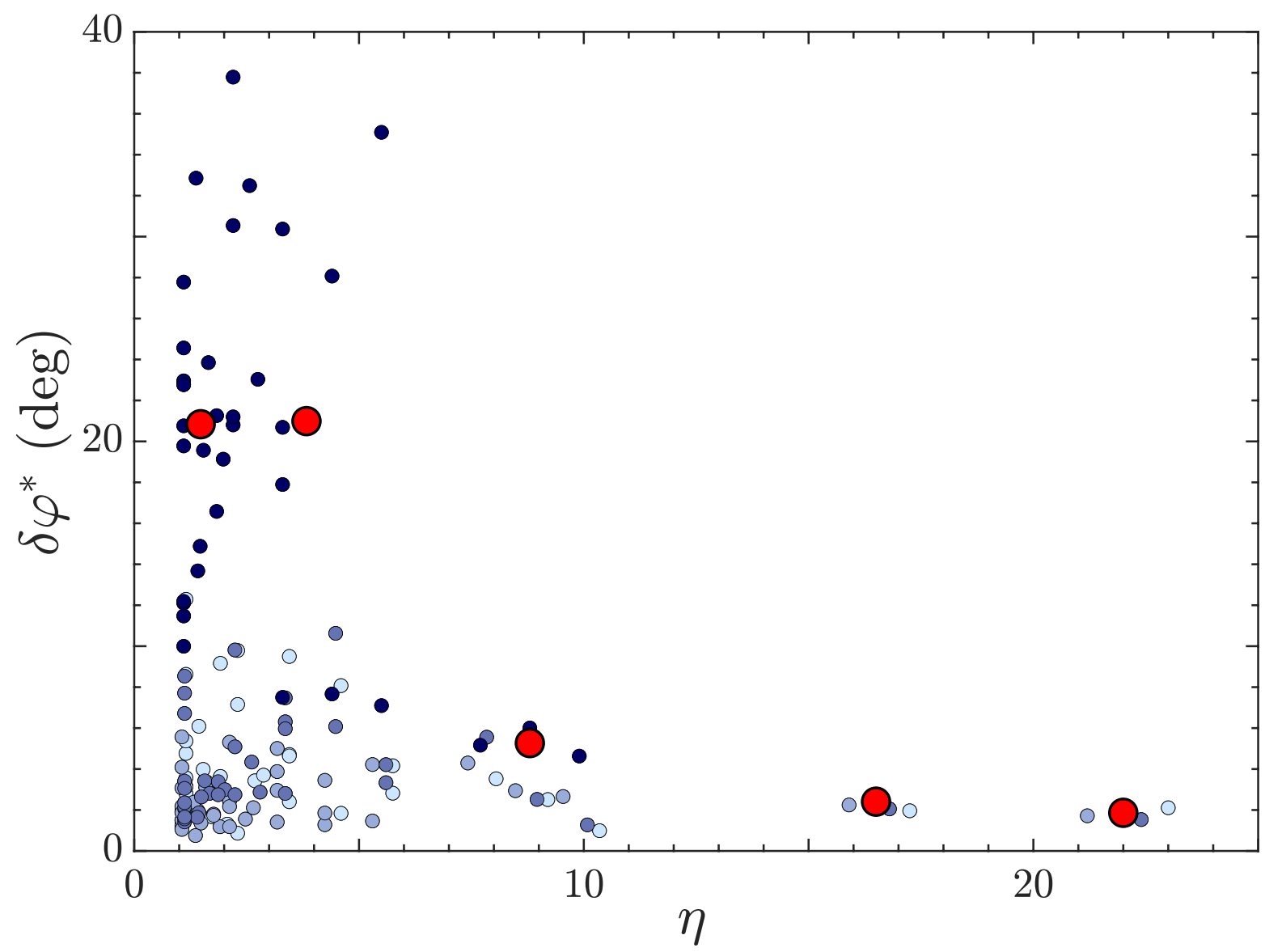

FIG. S5. Standard deviation of swimming direction $\delta\varphi^*$ among the cell population for all δ (color-coded from light to dark blue for increasing δ, as Fig. 1(c) of main text). For the largest angle $2\delta \approx 135°$ (dark blue) the standard deviation increases drastically as $\eta \to 1$ (for $\eta \lesssim 5$). This is shown by the red circles, obtained by binning the data with similar values of η.

S3. LIGHT-SWITCHING EXPERIMENTS

A. Obtaining the switch time t_0

The experiments were performed at a frame rate of 20 fps, using the same 4× objective as for experiments above. Movies were recorded for a period of 20 seconds, within which the light was switched manually at $\sim$ 10s after the start of the recording. To determine the video frame corresponding to the switching of the light, and hence the switch time t_0, we located the jump in total intensity of the video frames that arises from the very small intensity difference of the two light sources. Fig. S6 shows a typical intensity trace with such a jump.

B. Dispersion of initial value of $\sin\theta$ in light-switching experiments

In the light-switching experiments, the angle of swimming of the cells at the switch time t_0 does not correspond exactly to the light propagation direction $\hat{\mathbf{v}}_+$. This is a consequence of the helical motion of the cells that makes their orientation oscillate around the average direction $\hat{\mathbf{v}}_+$. Typical distributions of orientations before the switch are shown in Fig. S7 for $I_+ = 1.8\text{W/m}^2$ ($i_+ = 5\text{mA}$) (panel a) and $I_+ = 63.1\text{W/m}^2$ ($i_+ = 100\text{mA}$) (panel b). In order to compare with the theory (Fig. 2b and Eq. (5) of the main text), we need to take into account this variation in orientation at t_0, which makes the average value $\langle\sin\theta(T_0)\rangle$ larger than $-\sin 2\delta \approx -0.94$. The width of these distributions decreases as the light intensity increases (Fig. S7), consistent with the ordering of the curves in Fig. 2(b) of the main text at the switch time t_0.

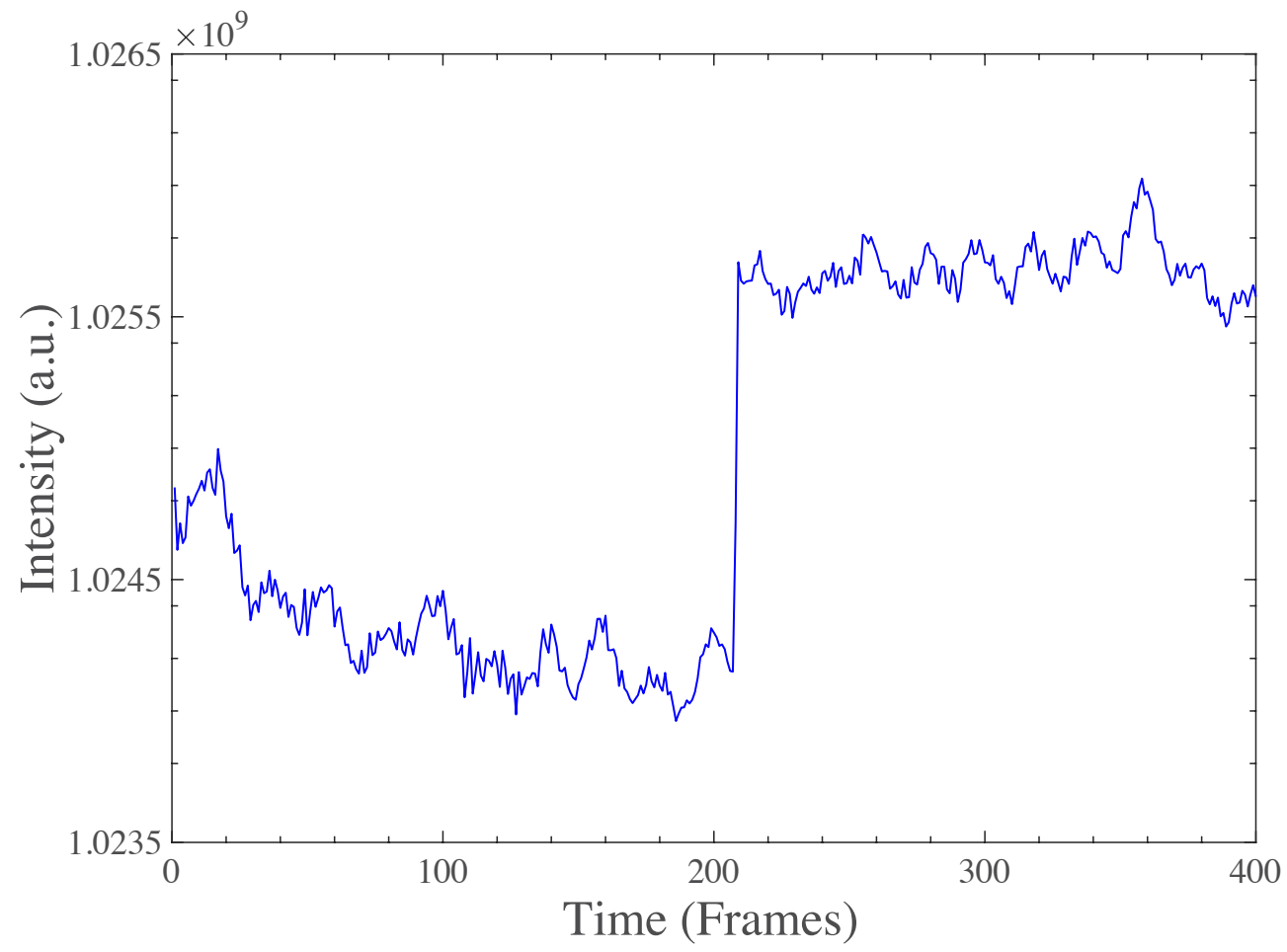

FIG. S6. Total frame intensity over the duration of an experiment for $i_+ = i_- = 50\,\mathrm{mA}$.

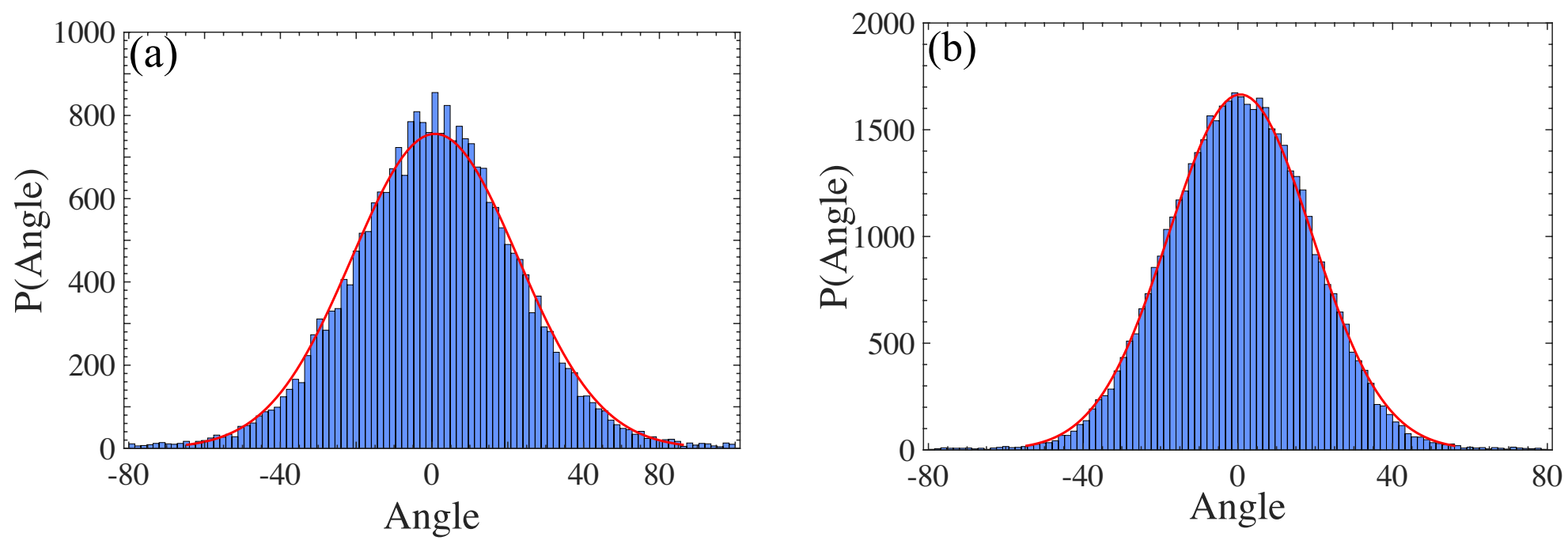

FIG. S7. Distribution of swimming directions prior to the switch time t_0 in light-switching experiments. The distributions are centered around the direction of light propagation $\hat{\mathbf{v}}_+$. (a) A Gaussian fit (red curve) at low intensity $I_+ = 1.8\mathrm{W/m^2}$ ($i_+ = 5\mathrm{mA}$) yields the average angle $\mu = 0.78^\circ$ and standard deviation $\sigma = 21.89^\circ$. (b) At the larger intensity $I_+ = 63.1\mathrm{W/m^2}$ ($i_+ = 100\mathrm{mA}$), $\mu = 0.63^\circ$ and $\sigma = 18.52^\circ$.

S4. BIFURCATION

A. Calibrating light intensity

For the bifurcation experiments at large angles 2δ between the lights, we require the intensity of each light to be exactly the same. Otherwise, there would be a strong asymmetry in the distribution of orientations $Q(\varphi)$ because cells are highly sensitive to even minute differences between the two light intensities.

To ensure that η was as close to 1 as possible, we calibrated the two LEDs using a Spectra Pen Mini (Photon System Instruments). A custom stage was built to hold the sensor at its center, exactly where the cells are imaged in the Petri dish. We then adjusted the electrical current sent to one of the lights to match exactly the intensity of the second light. In these experiments we used $I_\pm = 3.7 \pm 0.1\mathrm{W/m^2}$, which led to the distribution $Q(\varphi)$ in Fig. 3b that is quite symmetric, although a slight asymmetry persists ($N_{\varphi>60^\circ}/N_{\varphi<-60^\circ} = 1.55$). We surmise the asymmetry comes from a tiny remaining intensity difference between the lights, below the precision of our light power-meter ($0.1\,\mathrm{W/m^2}$).

B. Calculating the distribution of swimming directions $Q(\varphi)$

To obtain a detailed quantification of the swimming direction of the cells, we divide each trajectory into segments consisting of 5 full rotation periods (60 frames = 3s). From these segments we calculate the local angle (φ) with respect to the average direction ($\hat{\mathbf{v}}_+ + \hat{\mathbf{v}}_-$), yielding the distribution of orientations ($Q(\varphi)$, Fig. 3(b) in the main

text). The same analysis was performed on the simulated trajectories (Fig. 3(d) in the main text).

C. Eyespot imaging

In order to determine the distribution of eyespot locations in a population of *C. reinhardtii*, we first immobilized the cells on a microscope slide coated with polylysine (Electron Microscopy Sciences, #63410-01) by placing a 12 μL droplet onto the slide and covering it with a coverslip. We then combined the information obtained from three different types of imaging with a 40× objective (Olympus, UPlanSApo, 40×/0.95): brightfield microscopy, reflection microscopy, and epi-fluorescence microscopy (methods detailed below). Brightfield imaging (Fig. S8(a)) was used to characterize the cell shape. Reflection microscopy with an orange (580nm) light (Fig. S8(b)) was used to image the eyespot as a bright spot making use of the highly reflective properties of the carotenoid layer of the eyespot at this wavelength. Epi-fluorescence microscopy was finally used to image the chloroplast and obtain the polarity of the cells. Acquiring about 20 images with these methods, we obtained statistics of the eyespot location of ~ 200 cells. A superposition of the three types of images is shown Fig. S8(f) with the brightfield image in grey, the eyespot in red and the chloroplast in green.

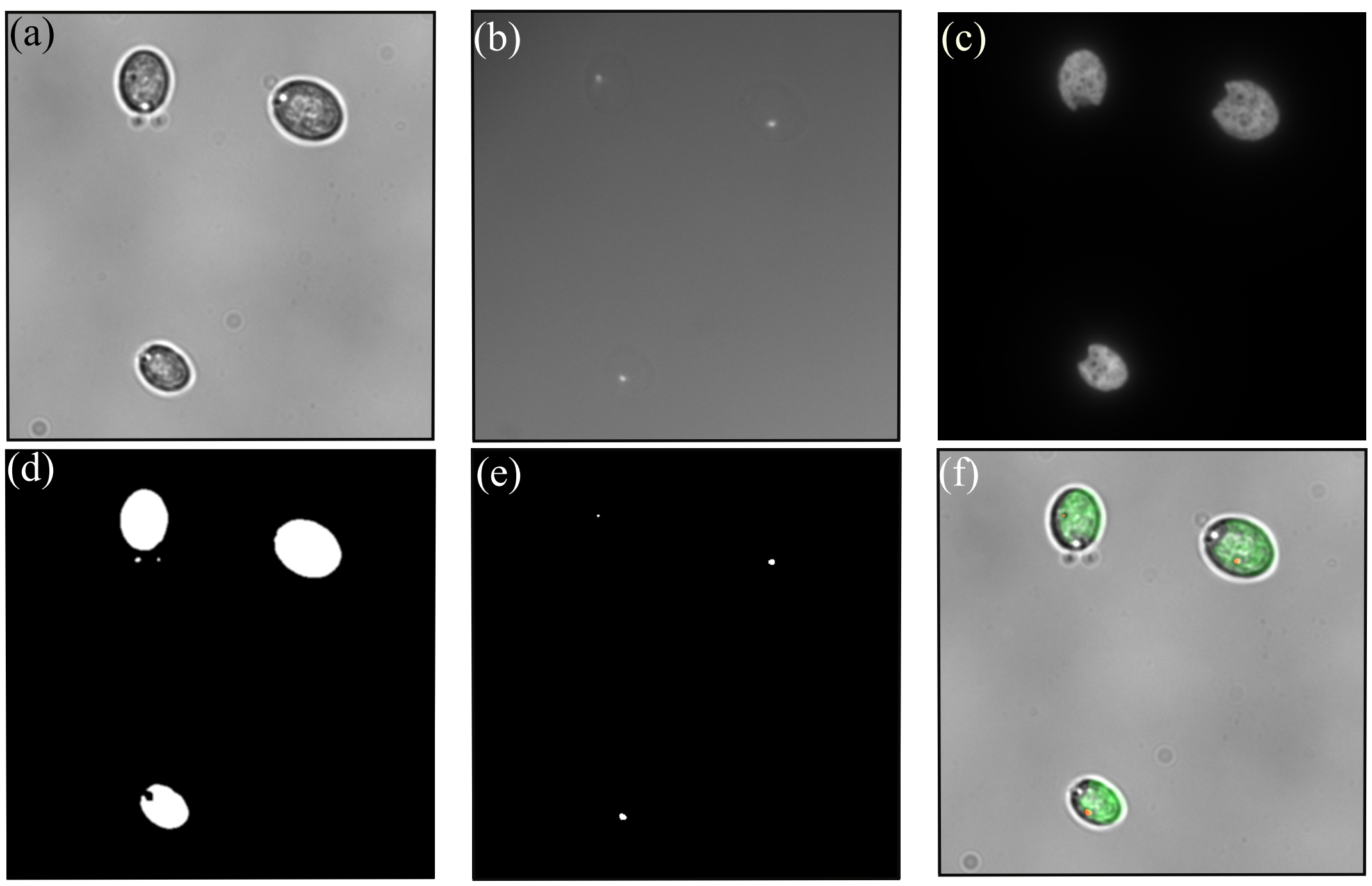

FIG. S8. Determining the eyespot location. (a) Brightfield image of the cell bodies, (b) eyespots (bright spots) in reflection microscopy, (c) chloroplast visualized using epi-fluorescence microscopy of the chlorophyll molecules, (d) binarized image of panel a, (e) binarized image of panel b, and (f) merged image of panels a, e and c in gray, red and green color respectively.

The brightfield images were binarized, followed by dilation and erosion to obtain ellipsoidal cell bodies (Fig. S8(d)) from which we extracted the centroid, the major and minor axis, the cell area (to filter out cells too close to each other to be detected as single cells) and the bounding rectangle of the features. The images from reflection microscopy were also simply binarized (Fig. S8(e)) to extract the eyespot position. From the information of the eyespot position and the major axis of the cells we obtained the distance of the eyespot from the cell equator, without knowledge of the direction (i.e. closer to the flagella or further away). This missing information was obtained from the epi-fluorescence images of the chloroplast which makes a U-shape at the back of the cells (Fig. S8(c)). Extracting the center of mass of the fluorescence signal allowed us to know the polarity of the cells. To do so we applied the binarized brightfield images as masks to the epi-fluorescence images, then cropped around each cell (using the bounding rectangle) and finally computed the center of mass of the fluorescence signal.

Details of the imaging methods. All images were acquired on an Olympus IX83 inverted microscope equipped with a Hamamatsu Orca Fusion-BT camera (C15440-20UP).

Brightfield. Brightfield images were captured using a red filter at 624 nm (BrightLine, FF01-624/40-25) to ensure

cells remained still by avoiding phototactic responses.

Reflection microscopy. White light from a Lumencor SOLA-VN LED source was sent through the back of the microscope into a filter cube containing an excitation filter at 580nm (BrightLine, FF01-580/60-25) and a glass slide acting as a semi-reflective surface to redirect light towards the sample and let the reflected light from the eyespots to reach the camera.

Epi-fluorescence microscopy. White light from a Lumencor SOLA-VN LED source was sent through the back of the microscope into a filter cube containing an excitation filter at 447nm (BrightLine, FF02-447/60-25), a dichroic mirror at 605nm (Thorlabs, DMLP605R) and an emission filter at 697nm (BrightLine, FF01-697/58-25) to collect the fluorescence signal of the chlorophyll molecules in the chloroplast.

S5. COLLIMATED LIGHTS

The experimental setup has been designed to have the least possible angular dispersion of the light propagation direction (and therefore light intensity gradients) within the field of view. The small field of view (3.7 mm×3.7 mm) is placed exactly at the center of the Petri dish where light rays are almost not altered by the air/Petri dish/water interfaces. Indeed the very center of the beam of each collimated LED arrives perpendicularly to the edge of the Petri dish (see Fig. 1a) and its direction is therefore not altered by these interfaces. However light rays away from the center of the beam will be deviated and refocused towards the field of view. From a simple ray optics calculation, using the Snell-Descartes law of refraction, we can estimate that within the field of view, the angular dispersion of the light rays is at most ±6°. In addition to this theoretical argument, we have performed fluorescence imaging of the field of view, adding fluorescein to the water, using the collimated LEDs as the excitation lights and collecting the light emitted by the fluorescein. These images demonstrate that there is a good uniformity in the intensity profiles within the field of view, as shown in Fig. S9 below. In all cases we can notice slight intensity gradients, which are always in the same directions, and therefore uncorrelated with the light direction of the LEDs. We believe this feature comes from the imaging system (most notably, the objective). In these profile the standard deviation of the light intensity is always below 4% (values given in the plots in Fig. S9), while the maximum and minimum intensity values deviate by less than 10% from the average values.

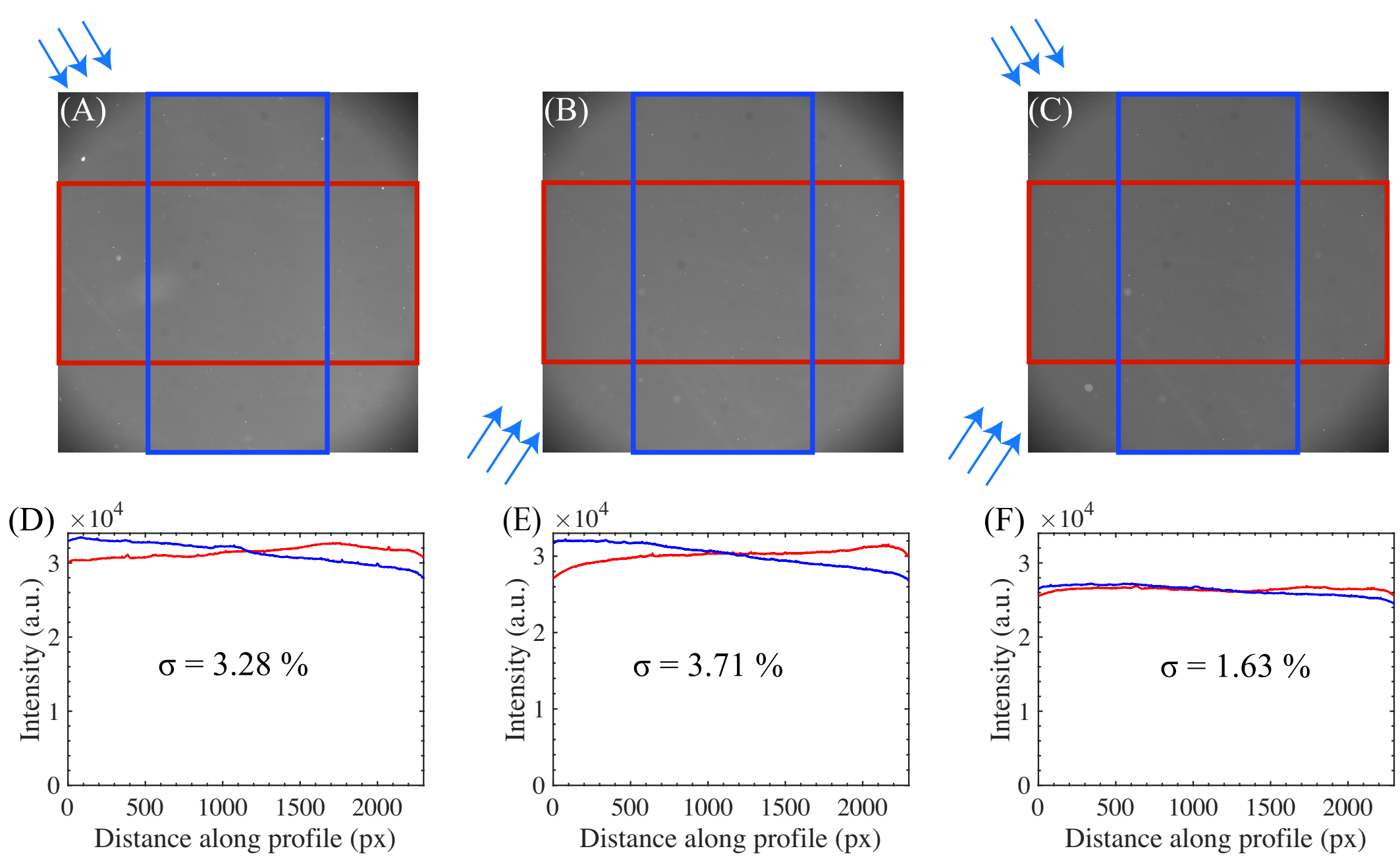

FIG. S9. A-C) Fluorescence images for LED 1 (A), LED 2 (B) and both lights (C). E-F) Corresponding intensity profiles along two perpendicular directions depicted by the red and blue ROI.

S6. CAPTIONS FOR SUPPLEMENTARY MOVIES

Supplementary Movie 1: Cells following the tangent law, at various values of $\eta = 1.2, 2.9, 16.6$. Video captured at 4x magnification, 20fps, played in real time. The green arrow depicts the average swimming direction of the population.

Supplementary Movie 2: On changing the light, we see the fast turns that the cells make when the light is switched from $\hat{\mathbf{v}}_+$ to $\hat{\mathbf{v}}_-$. Video captured at 4x magnification, 20fps, played in real time.

Supplementary Movie 3: At bigger angle of $2\delta = 162^\circ$ and $\eta = 1$ different subpopulations of cells emerge: cells following either light, cells following the tangent law and cells switching stochastically between these two. Video captured at 4x magnification, 20fps, played in real time.
